# Supplementary material for: Are carbon emissions trading and green financial instruments synergistic? -Comprehensive quantitative research based on content analysis
Source: PLoS One. 2024 Mar 7;19(3):e0298601. doi: 10.1371/journal.pone.0298601 (PMC10919601; doi:10.1371/journal.pone.0298601)
Supplement: S1 Data — (ZIP) [file pone.0298601.s001.zip › Data/地级市Z_F工作报告.zip!/╘╞─╧/┴┘▓╫╩╨/└┤╫╘╣1⁄2╓┌║┼í╛┬φ┐╦╩2╛▌═°í┐_2016.docx]

各位代表：

　　现在，我代表市人民政府，向大会报告工作。请各位代表连同《临沧市国民经济和社会发展第十三个五年规划纲要（草案）》一并审议，请市政协委员和列席人员提出意见。

一、2015年和“十二五”时期经济社会发展回顾

　　2015年，是临沧经济社会发展极其艰难、极不平凡的一年，也是困难最多、挑战最大的一年。在市委的坚强领导下，在市人大及其常委会的法律监督、工作监督和市政协的民主监督下，市人民政府深入贯彻落实习近平总书记系列重要讲话精神和对云南工作重要指示精神，贯彻落实李纪恒书记、陈豪省长视察临沧重要讲话精神，主动服务和融入国家“一带一路”、孟中印缅经济走廊，特别是辐射中心建设战略，努力适应经济发展新常态，把握经济发展新常态，引领经济发展新常态，坚持稳中求进、积极有为工作总基调，保持定力、攻坚克难，奋力工作、开拓创新，团结和依靠全市各族人民，正确应对和妥善处置缅北果敢“2·09”军事冲突，快速反应，迅速组织沧源“3·01”地震灾害的抗震救灾和恢复重建工作，全力以赴稳增长、调结构、促改革、惠民生、防风险，着力抓好30项重要指标和“四个一百”重大项目，经济增长低开高走、逐季向好，实现稳中有升，主要经济指标超额完成“十二五”目标。全市政治稳定、经济发展、民族团结、边境安宁、社会和谐。

　　（一）采取有效的有力措施，全力以赴稳增长。强化经济形势研判和经济运行调控，及时出台稳增长29条政策，生产总值增长10%，排名全省第5位。主动争取国家和省的资金、项目、政策支持，一批事关临沧长远发展的重大项目挤入国家、省规划盘子。“四个一百”重点项目扎实推进，18个重大项目列入国家和省PPP项目库，16个项目获国家专项建设基金支持。规模以上固定资产投资增长29.1%，规模和增速排名全省第5位。扎实开展服务企业年活动，实行领导挂钩联系服务重点企业、重点项目，设立工业企业应急互助资金，增强要素保障，降低运营成本，保障企业正常生产经营，鼓励支持企业上市。规模以上工业增加值增长10.6%，排名全省第4位。加强财税工作，一般公共预算支出跨过200亿元大关。改革投融资体制，加强融资平台建设，推动银政企合作，吸引社会资本参与投资，金融服务实体经济力度持续加大。加强市场调控，保持房地产市场稳定。城镇常住居民人均可支配收入增长8.7%，排名全省第5位；农村常住居民人均可支配收入增长12%，排名全省第2位；社会消费品零售总额增长12.6%，排名全省第2位；城镇登记失业率3.8%。

　　（二）狠抓“五网”基础设施建设，高速公路铁路建设取得历史性突破。启动“五网”建设5年大会战，临沧机场高速公路、玉溪至临沧高速公路、大理至临沧铁路顺利开工，铁路、高速公路建设实现“零”的突破。临沧至清水河铁路、临沧至普洱铁路完成预可研，临沧至清水河高速公路前期工作加快推进。临沧机场改扩建一期工程基本完工，沧源佤山机场建设进展顺利，孟定机场前期工作加快推进，凤庆、永德通用机场场址通过专家评审。耿马灌区工程规划报告通过水利部审查，澜沧江小湾提水供水工程前期工作快速推进。康家坝中型水库和3件小（一）型水库竣工验收，5件小（一）型水库开工建设，完成“五小水利”工程2万件。完成库塘蓄水3.3亿立方米，解决16万农村人口饮水安全问题。南汀河段治理工程加快推进，完成23件中小河流治理。综合治理水土流失270平方公里。城市综合体建设进展顺利，城镇道路、供排水、垃圾污水处理等基础设施不断完善。“洁净临沧”行动计划深入实施，城乡环境明显改善。500千伏博尚变主变工程顺利投产。“智慧临沧”建设加快，4G网络和有线电视实现乡（镇）全覆盖。

　　（三）强力推动对缅合作，沿边开放实现突破。立足区位优势，全力做好沿边开放这篇大文章。临沧市友好代表团成功访问缅甸，分别与缅甸中央和地方军政主官进行会谈，就推动基础设施互联互通、通关便利化、经贸和人文交往与缅甸官方和民间两个层面深化交流，着力解决好人财物“三通”问题。临沧边境经济合作区建设加快，作为全国政协第三十六次双周协商座谈会主要协商内容。临沧边境经济合作区和孟定国家级口岸被国务院纳入沿边重点开放地区支持名录。推动援建滚弄大桥工作取得积极进展。清水河口岸迁址新建项目开工。大额人民币现钞出入境携带证管理试点工作进展顺利。清水河口岸持护照通行、国家重点坚果实验室、芒卡进口矿产品国检监管区和清水河粮食进口指定口岸获批。第五届临沧边境经济贸易交易会成功举办。全市招商引资实际到位资金830.4亿元，增长24.3%，其中省外到位资金620.6亿元，增长21.3%，总量排名全省第4位。

　　（四）充分发挥生物资源优势，特色产业建设持续推进。传统优势产业巩固提升，新增高原特色农业产业化基地127.2万亩。获得2018年世界坚果大会举办权。农村发展综合体建设加快，新增农民专业合作社52个、农业龙头企业11户。粮食行政首长负责制全面落实，粮食总产量达104万吨，肉类总产量28万吨，水产品产量6.4万吨。第一产业实现增加值145.3亿元，增长6.1%。临沧工业园区、云县新材料光伏产业园区列入全省拟培育的百亿元以上园区，凤庆滇红生态产业园被列为全国滇红茶知名品牌创建示范区、全省第一批生物产业示范基地、省级农业科技园区。新增28户规模以上工业企业。实施规模以上工业项目445个，完成工业固定资产投资147.8亿元，增长23.1%；民营经济不断壮大，实现增加值220.7亿元，增长10.6%。第二产业实现增加值169.8亿元，增长12.5%。沧源国际旅游度假区建设加快，全市接待国内外旅游者增长36%，旅游总收入增长53.8%。“互联网+电子商务”得到推广，乡村电子商务平台建设取得进展；工业企业电子商务、物流快递业高速增长。成功举办第三届亚洲微电影艺术节，临沧知名度、美誉度不断提升。第三产业实现增加值187亿元，增长9.9%。

　　（五）全面发展社会事业，民生保障水平不断提高。启动“27241”脱贫攻坚大会战，以农村危旧房改造为抓手，实施精准脱贫，认真开展“挂包帮”“转走访”工作，扎实推进易地扶贫搬迁三年行动计划和扶持彝族支系俐侎人发展三年专项规划；完成农村危房拆除重建4.26万户、美丽村庄提升113个，减少贫困人口6万人。景谷“10·7”地震受灾民房恢复重建全面完成，受灾群众全部搬入新居。沧源“3·01”地震恢复重建有序推进。学前教育加快发展，义务教育均衡发展。普通高考再创佳绩，一本上线率居全省第4位。滇西科技师范学院批准成立，首批本科生入学就读。文化惠民措施有效落实，群众性体育活动全面开展，广播电视覆盖率达98.3%。医疗卫生体系不断健全。临沧市人民医院新院正式开诊。城镇新增就业2.5万人，农村新增转移就业100.3万人次。按期完成机关事业单位工资制度改革补发兑现工作。城乡居民实行统一的基本养老保险制度，各项保险参保总人数达193.7万人。关注社会弱势群体，46万人纳入城乡低保。食品药品安全监管工作全面落实，安全生产形势稳中向好。法律服务和法律保障更加充分。第三轮禁毒防艾人民战争深入开展，社会治安防控体系进一步完善。2015年10件惠民实事全部完成。

　　（六）妥善处置“2·09”事件，全力维护边境稳定。在党中央、国务院和省委、省政府的坚强领导下，讲政治、顾大局，积极妥善、正确处置“2·09”事件，维护了国家尊严和领土安全，维护了人民群众的生命财产安全，确保了边境大局平稳可控，社会和谐稳定，群众生产生活秩序正常，经济社会持续健康发展。我们准确把握形势，充分发挥地缘人缘优势，积极开展人道主义救助，进一步了解缅甸、熟悉缅甸，进一步夯实了与缅甸友好交流、经济合作的基础。临沧人民为国家安全、守边固土作出了贡献，为党和国家分了忧、争了光，全市各族干部群众感到无比骄傲和自豪。

　　（七）认真践行“三严三实”要求，政府自身建设明显推进。扎实开展“三严三实”和“忠诚干净担当”专题教育。切实加强服务政府、责任政府、法治政府、廉洁政府建设。政府职能进一步转变，市政府工作部门调减为32个，市级行政审批事项缩减到200项，成为全省行政审批事项最少的州市之一。全面推行政府权力清单和责任清单制度。加强财政资金管理、公共资源交易制度建设，财政预决算、“三公”经费、重大项目实施、社会公益事业建设等领域的政务公开力度持续加大。民生资金实现电子监管。全面推行“五级联动”机制，群众诉求表达渠道更加畅通。健全完善市、县、乡、村四级政务服务中心体系，企业和群众办事更加方便。强化权力制约和监督，自觉接受人大及其常委会和政协的监督，接受新闻舆论和社会公众监督，进一步加强行政监察和审计监督，认真落实主体责任和监督责任，党风廉政建设和反腐败工作取得新成效。

　　去年工作取得的成绩，保证了“十二五”圆满收官。“十二五”时期是临沧发展最快的时期之一，经济社会各项事业发展取得了显著成效，为“十三五”顺利开局和与全国全省同步全面建成小康社会奠定了坚实的基础。

　　——过去五年，综合经济实力显著增强。我们始终把发展作为第一要务，在实现高速增长中不断提高经济增长质量和效益。“十二五”末，全市生产总值突破500亿元大关，达502.1亿元，年均增长13.3%，人均生产总值20077元，年均增长12.5%；规模以上固定资产投资729.2亿元，年均增长37.2%；城镇常住居民人均可支配收入21225元，年均增长11.8%；农村常住居民人均可支配收入8063元，年均增长18.8%；一般公共预算收入38.1亿元，年均增长21.3%；一般公共预算支出202.5亿元，年均增长17.4%；社会消费品零售总额154.6亿元，年均增长16.3%；外贸进出口总额39.2亿元，年均增长23.7%；金融机构存款余额510.6亿元，年均增长15.8%，贷款余额432.1亿元，年均增长20.8%。

　　——过去五年，基础设施建设快速推进。我们始终把高速公路、铁路建设作为重中之重，举全市之力高位推动、强力推进，铁路、高速公路建设取得历史性突破，实现了临沧人民百年梦想。8条（段）二级公路全面建成，全部乡（镇）通油路，建制村通畅率达84%。农田水利基础不断夯实。完成中低产田地改造150.7万亩；8件中型、18件重点小（一）型骨干水源工程开工建设，147件小（二）型水库除险加固全面完成，累计建成中小型水库248座，“五小水利”工程11万件，新增库容1.6亿立方米，耕地有效灌溉保证率达45%；85万人农村饮水安全问题得到解决。城乡电力通信保障能力明显提升。累计完成地方水电总装机100万千瓦，太阳能光伏发电和城市天然气综合利用稳步推进，农村人口用电覆盖率和农村居民户表改造率均达100%；乡镇光缆、有线宽带和建制村无线通信网络实现全覆盖。

　　——过去五年，对外开放不断扩大。我们始终坚持发挥沿边区位优势，加快推进沿边开放。国家级临沧边境经济合作区获得批准并加快建设，累计实施项目435个，完成固定资产投资591.9亿元，入驻企业达201户，招商引资实际到位资金708.6亿元。口岸通关便利化取得进展，清登二级公路建成通车，中缅边境3条国际旅游线路获批开通，连续5年成功举办临沧边境经济贸易交易会。与缅北地区合作开发特色产业基地120万亩，29户企业在缅北地区投资经商，57户缅籍企业落户临沧。一批国企、外企和有实力民企入驻临沧发展。全市累计利用外资比“十一五”增2倍，招商引资实际到位资金连年保持高速增长。与斯里兰卡加姆波勒市、俄罗斯杜布纳市等友好城市交流不断深化。

　　——过去五年，产业转型升级步伐加快。我们始终立足资源、发挥优势、突出特色，努力转变发展方式。三次产业比例由32.9:35.1:32调整为29:33.8:37.2。累计建成高原特色农业产业化基地2102万亩，澳洲坚果面积全国第1，咖啡面积全国第2，甘蔗面积和蔗糖产量全省第1，茶叶产量全省第1，核桃面积全省第2，橡胶面积和产量全省第3。粮食生产实现八连增。加快培育农村新型市场主体，发展农民专业合作社1721个，家庭农场976个，农业龙头企业130户。工业园区实现“一县一园”全覆盖。规模以上工业企业达142户。亚洲微电影艺术节、“摸你黑”狂欢节等节庆活动综合效应凸显。实现接待国内外旅游者年均增长23.2%，旅游总收入年均增长35.3%。

　　——过去五年，生态文明建设稳步推进。我们始终注重生态文明建设，不断提高可持续发展能力。加快建设临沧国家可持续发展实验区和全国低碳国土试验区，开展“七彩云南·秘境临沧”保护行动，扎实推进南汀河流域生态综合治理，重点生态功能区保护全面加强，自然保护区面积达303.4万亩，境内分布的原始生态系统类型和野生动植物物种得到有效保护。累计治理水土流失1200平方公里，完成退耕还林208.8万亩，新造林724.4万亩，森林覆盖率提高到67.5%。空气质量优良率达100%。县级及以上集中式饮用水水源地水质100%达到国家标准。临沧被省政府命名为省级园林城市，获得“中国恒春之都”、“中国十佳绿色城市”、“中国最具茶文化魅力城市”、“云南最佳生态州市”等荣誉称号。

　　——过去五年，城乡面貌发生较大变化。我们始终以人为核心，不断加快新型城镇化步伐。临沧中心城市缅宁大道、玉龙湖、南汀河临沧城段治理、临沧花果山城市森林公园等一批项目建成。建设保障性住房6.3万套。城镇建成区面积比2010年增加24平方公里，建成区绿化覆盖率达36.5%，城市垃圾无害化处理率和污水处理率分别达到93%和88%，城市管理水平明显提高。住房公积金缴存比例提高到12%，住房资金保障能力不断增强。农民进城工作有序开展，46.8万农业人口转变为城镇居民，城镇化率达36.86%。美丽家园建设扎实推进，沧源“佤山幸福工程”圆满完成，完成旧村改造2601个，旧房改造19.3万户，美丽村庄提升223个。

　　——过去五年，各项改革扎实有效推进。我们始终把深化重点领域、关键环节改革作为推进经济社会发展的强大动力。完成新一轮政府机构改革，政府职能加快转变，市级行政审批事项大幅精简。预算制度改革全面推进，“营改增”试点范围不断扩大。“三证合一、一照一码”登记制度改革全面启动。农村土地、集体林权、水利、农垦、供销社、粮食流通等领域改革不断深化。沿边金融综合改革取得有效进展。县级公立医院改革、教育领域综合改革、生态文明体制改革稳步推进。

　　——过去五年，民生保障水平持续提高。我们始终把人民群众对美好生活的向往作为政府工作的目标。财政资金中民生支出达70%以上。教育事业加快发展，校安工程全面完成，新建校舍200万平方米。农村义务教育学生营养改善计划全覆盖。普通高考连续多年排名全省前列。覆盖城乡的公共卫生和医疗卫生服务体系基本建立，“看病难、看病贵”问题有效缓解。计划生育国策全面落实，人口自然增长率控制在7‰以内。就业创业政策措施不断完善，城乡就业质量和水平不断提高，城镇登记失业率控制在4.5%以内。城乡居民养老保险达127.9万人，城镇职工基本医疗、基本养老、失业、工伤和生育保险覆盖面不断扩大，社会保险、社会救助、社会福利、优抚安置等保障水平不断提高。文化遗产保护和公共文化服务体系建设取得明显成效，群众性文化体育活动广泛开展。妇女儿童发展规划实施成效突出。公民具备基本科学素质比例达3.36%，科技进步对经济增长的贡献率达46.5%。安全生产形势持续好转，食品、药品安全监管得到加强。“民族团结进步边疆繁荣稳定示范区”、“兴边富民”、扶持人口较少民族发展和边疆解“五难”工程扎实推进，累计减少贫困人口50万人。党的宗教政策全面贯彻落实，社会创新和治理进一步深化，“平安临沧”建设深入推进。统计、档案、气象、侨务、人防等工作扎实推进。

　　各位代表，过去五年，全市经济社会发展取得的成绩，靠的是以习近平同志为核心的党中央的坚强领导，靠的是中国特色社会主义制度的优越性，靠的是省委、省政府的倾力关心支持，靠的是市委带领全市广大干部和各族人民的团结奋斗。在此，我谨代表市人民政府，向全市各族群众，向人大代表、政协委员、各民主党派、工商联和无党派人士、各人民团体、社会各界人士，向驻临人民解放军和武警部队官兵，向所有关心支持临沧发展的港澳同胞、台湾同胞、海外侨胞和国际友好人士，表示衷心的感谢！

　　在充分肯定成绩的同时，我们也清醒地认识到，临沧仍然是典型的欠发达边疆民族地区，推进发展面临着诸多困难和问题：“五网”基础设施“短板”制约突出，基础设施对经济发展的支持功能较弱；沿边开放水平不高，“人财物”流通不畅；产业发展层次不高，资源型产业特征明显，产业转型升级任务艰巨；发展不平衡，农村贫困人口多、贫困程度深，基本公共服务的能力和水平亟待提高；科技创新能力较弱，人才支撑明显不足；安全生产、食品药品安全、社会治安、边境管控压力仍然很大；干部的思想水平、工作能力、服务意识和工作效率有待提高，政府职能转变还不到位。我们将正视这些困难和问题，迎难而上，开拓创新，勇于担当，把各项工作做得更好，不辜负全市人民的重托。

二、“十三五”时期的目标任务

　　“十三五”时期是临沧与全国全省同步全面建成小康社会的决战期、决胜期。我们面临难得的发展机遇，具备加快发展的条件，完全有可能也必须实现跨越发展。党的十八大以来以习近平同志为核心的党中央提出的一系列治国理政新思想新战略，是全党在建设中国特色社会主义实践中对马克思主义理论的创新和发展，是中国特色社会主义理论体系的最新成果，是全党集体智慧的结晶，为我们在新的历史条件下战胜一切艰难险阻提供了科学的理论指导和行动指南；习近平总书记对云南发展的“三个定位”，高度契合临沧市情和发展需要，为我们推进跨越发展指明了前进方向；国家加快实施“一带一路”和孟中印缅经济走廊战略，大力支持西部地区、边境民族地区、革命老区、贫困地区加快发展，大力支持云南建设面向南亚东南亚辐射中心，为我们推进跨越发展提供了战略支撑；李纪恒书记、陈豪省长等省委、省政府领导多次深入临沧，对临沧工作高度重视，将临沧列为五出境通道之一，在基础设施建设、沿边对外开放、脱贫攻坚等方面给予大力支持，极大地提振了全市各族干部群众干事创业的信心和决心，为我们推进跨越发展注入了强大动力；“十二五”时期全市建成一批重大基础设施和重大产业项目，经济社会取得长足发展，为我们推进跨越发展奠定了坚实基础；临沧无可替代的区位优势、开放优势、资源优势和战略地位逐步被国家和省级层面认知、认可，只要我们积极主动，融入和服务国家战略，在对缅开放上争当马前卒、排头兵，主动作为，取得突破，临沧完全有可能成为辐射中心建设的主要通道、基地和平台，成为云南、西南乃至全国面向印度洋开放的重要通道、枢纽和节点。

　　“十三五”时期，市人民政府要认真贯彻市委《关于制定国民经济和社会发展第十三个五年规划的建议》提出的指导思想：高举中国特色社会主义伟大旗帜，全面贯彻党的十八大和十八届三中、四中、五中全会精神，以马克思列宁主义、毛泽东思想、邓小平理论、“三个代表”重要思想、科学发展观为指导，深入贯彻习近平总书记系列重要讲话和考察云南重要讲话精神，按照省委九届十二次全会、市委三届八次全会的安排部署，坚持全面建成小康社会、全面深化改革、全面依法治国、全面从严治党的战略布局，贯彻创新、协调、绿色、开放、共享五大发展理念，主动服务和融入国家发展战略，坚持发展是第一要务，突出修好路、兴水利、改危房、强产业、脱贫困、建生态、扩开放等重点工作，统筹推进经济建设、政治建设、文化建设、社会建设、生态文明建设和党的建设，努力在民族团结进步示范区、生态文明建设排头兵、面向南亚东南亚辐射中心建设中走在全省前列，实现跨越式发展，确保与全国全省同步全面建成小康社会，为谱写好中国梦临沧篇章奠定更加坚实的基础。

　　“十三五”时期临沧要实现经济社会跨越发展，必须坚持适应、把握、引领新常态，实现逆势上扬、弯道超车；必须坚持以“改革、创新、开放”为动力，用改革转换发展新动力，用创新拓展发展新空间，用开放提升发展新高度；必须坚持以“协调、绿色、共享”为可持续发展的重要保障，用协调解决发展的不平衡，用绿色保障发展的可持续，用共享体现发展的新成果；必须坚持以市场为导向，更加注重供给侧结构性改革，不断扩大有效投资，在推进一、二、三产融合发展中增加有效供给，全面提高发展速度、质量和效益。“十三五”发展的主要目标是：经济年均增长11%以上，城乡常住居民人均可支配收入增长12%左右，全市经济发展的质量和效益全面提升，四化水平全面提升，改革开放水平全面提升，民生保障水平全面提升，基础设施更加完备，产业结构更加合理，城乡环境更加宜居，生态环境更加优美，人民生活更加幸福。

　　实现“十三五”规划目标，政府工作必须紧紧围绕跨越发展这条主线，重点在八个方面实现重大突破：

　　（一）强力推进铁路高速公路建设，在“五网”建设上实现重大突破。坚决打好“五网”基础设施建设5年大会战，构建互联互通、功能完备、高效安全、保障有力的基础设施网络。加快建设以航空为先导，高速公路、铁路为重点，水运为补充的立体综合交通网络。机场高速、玉临高速和大临铁路建成通车；开工建设临清高速、临清铁路，并争取建成通车；启动建设大临高速、玉临铁路、沿边高等级公路、沿边铁路，争取形成实物工程量；推进清水河至缅甸腊戍的铁路和高等级公路境外段建设。到2020年，力争建成高速公路232公里，在建高速公路185公里；建成铁路96公里，在建铁路220公里，基本建成“飞燕型”陆路交通体系。全面完成建制村公路通畅工程，实现油路到乡、硬化路到村、村组公路通达。沧源佤山机场建成通航，完成临沧机场改扩建，力争开工建设孟定机场和凤庆、永德通用机场。加快澜沧江-湄公河国际航道向上游延伸，开通澜沧江航运通道。开工建设耿马灌区等一批重大水利工程，加快推进云县岔河等13件中型水库和40件小（一）型水库前期工作并力争开工建设，基本建成安全可靠的水资源保障网。耕地有效灌溉保证率达55%以上，县城以上城市供水、农村自来水普及率分别达100%和80%以上。加快建设高效安全骨干电网，继续推进农村电网升级改造，积极推进天然气综合利用，适度开发利用太阳能、风能、生物质能，基本建成保障有力、绿色安全的能源保障体系。积极融入全省“云上云”行动计划，强化云计算、大数据、“互联网+”等技术的应用，确保建制村100%通有线光宽带，实现4G网络城乡全覆盖，基本建成共享普惠、高速高效的信息化基础设施。实施好《临沧市重大建设项目工作目标考核奖惩办法》，规模以上固定资产投资年均增长20%以上。

　　（二）强力推进对缅开放合作，在辐射中心重要门户建设上实现重大突破。用辐射中心思想统领对外开放工作，以通为本，千方百计破解对缅开放“人财物”流通不畅问题，真正把临沧建成中国、云南对缅开放的主通道。落实“一轴两城三带”战略，把孟定建设成为全省对外开放的前沿，建设成为新兴口岸城市，建设成为区域性的国际化都市。加强口岸建设，推进清水河口岸持护照通行，推动区域性一体化通关，提升通关便利化水平。认真贯彻国家、省支持沿边重点地区开放政策措施，加快临沧边境经济合作区建设，争取设立跨境经济合作区、跨境旅游合作区、沿边自由贸易试验区和综合保税区、国际产能合作示范区、进出口加工区，建成面向南亚东南亚国家进出口商品集散和生产加工基地。推进沿边金融综合改革试验区建设，推动跨境人民币业务创新。发挥地缘优势，继续深化与缅北地区农业合作。加快对外贸易优化升级，推动边境小额贸易向一般贸易、加工贸易、转口贸易发展。外贸进出口年均增长15%以上，实际利用外资年均增长10%以上，对外投资有较大突破。以对缅开放带动全方位开放，深化友城合作，加强与泛珠三角、长三角等区域合作，全市招商引资实际到位资金年均增长15%以上。

　　（三）强力推进生物资源开发利用，在特色产业建设上实现重大突破。坚定不移走“两型三化”产业发展新路子，实施质量强市战略，打造产业特色品牌，推进三产融合发展，加快构建开放型产业体系，促进经济行稳致远、行快致远。提升传统生物产业，加快培育新兴生物产业，建成一批现代农业产业园，培育一批涉农企业“小巨人”。主动融入全省澜沧江开发开放经济带建设，打造澜沧江绿色发展经济带和南汀河生物产业经济带。创建全国坚果产业发展示范区，举办好2018年世界坚果大会。农业增加值年均增长5%以上。坚持工业主导，构建以农产品加工业为重点的绿色工业产业体系。创新园区管理体制机制，把园区建成全面推进工业化、城镇化、农业现代化、信息化的重要引擎。建成临沧工业园区、云县新材料光伏产业园区两个百亿元园区，加快建成一批专业化园区，促进工业集聚发展。把凤庆滇红生态产业园打造成全国滇红茶知名品牌创建示范区，把红茶产业培育成为世界知名、全国第一。加大企业扶持力度，每个县区每年培育升规企业5户以上。加快传统工业与信息产业融合发展，规模以上工业增加值年均增长14%以上。大力发展民营经济，民营经济增加值占GDP比重达55%以上。大力培育生态文化旅游，建成沧源国际旅游度假区，打造滇西南生态养生旅游圈核心，接待国内外旅游者年均增长20%以上，旅游业总收入年均增长25%以上。巩固发展传统服务业，积极发展快递物流、现代金融、健康养生、科技服务等现代服务业，不断扩大消费需求，社会消费品零售总额年均增长12%以上。

　　（四）加快推进城市工作，在城乡一体化发展上实现重大突破。实施区域发展总体战略，统筹空间、规模、产业三大结构，规划、建设、管理三大环节，改革、科技、文化三大动力，生产、生活、生态三大布局，政府、社会、市民三大主体，加快推进以人为核心的新型城镇化。着力构建“一轴两城三带”发展空间格局，以昆孟国际大通道临翔至清水河段为主轴，以临沧中心城区和孟定新城为重要支点，以沿边地区、沿澜沧江地区、沿南汀河地区为重点发展区域，积极培育新的增长极，辐射带动全市经济社会加快发展。着力构建“1+8”城市布局，提升城市综合承载能力，把孟定和各县城打造成各具特色、宜居宜业的美丽城市。保护坝区农田，集约节约利用城市建设用地，重视城市地下和地上基础设施建设。开展水生态文明城市、海绵城市、智慧城市建设，保护文化、传承文脉、塑造特色，提升城市品位。加快棚户区改造，推进产城融合，支持撤县设市、撤乡建镇。落实住房公积金制度。改革户籍制度，有序推进农业转移人口市民化。按照“望得见山、看得见水、记得住乡愁”的要求，用3年时间把乡镇建成“小而美、小而干净、小而宜居”的特色城镇。继续实施“美丽宜居乡村”建设和“洁净临沧”行动计划，挖掘文化内涵，展现地域特色，建成一批特色村庄、洁净村庄。到2020年城市建成区面积达110平方公里，城镇化率达46%以上。

　　（五）加快生态环境保护，在生态文明建设走前列上实现重大突破。推动形成绿色发展方式和生活方式，促进人与自然和谐共生，建成国家级生态文明市。健全生态文明制度体系，加快主体功能区建设，加强自然遗产保护，深入实施滇西南生物多样性保护建设工程，持续推进城乡造林，扩大森林、湿地等绿色生态空间比重，新建永德大雪山国家公园，国家森林城市建设通过考评，到2020年森林覆盖率达70%以上。加快生态综合治理，重视澜沧江、南汀河和城市过境河流治理，强化城乡饮用水水源地保护，重点区域水土流失得到有效治理，重点地区水生态环境恶化状况得到有效控制。按照“适度开发、循环发展”要求，增强林业发展活力，建设“绿色银行”。落实最严格的环境保护制度，推行领导干部自然资源资产离任审计，实施全民节能行动计划，注重高效利用资源。能源和水资源消耗、建设用地、碳排放和主要污染物排放总量得到有效控制。保持空气环境质量优良率达100%，县级及以上集中式饮用水水源地水质全面达标，农村卫生厕所普及率达95%以上，规模化畜禽养殖废弃物综合利用率达80%以上。

　　（六）加快民族地区发展，在民族团结进步示范区建设上实现重大突破。深入开展民族团结进步教育，使“三个离不开”和“各民族都是一家人，一家人都要过上好日子”的思想观念深入人心，落到实处。巩固和发展平等团结互助和谐的社会主义新型民族关系，不断加强各民族交流交融。把民族地区发展融入全市发展大局，加大对民族地区扶持力度，不让一个兄弟民族掉队，不让一个民族地区落伍。集中力量对贫困民族乡、民族村等特殊困难区域，布朗族、德昂族、景颇族3个人口较少民族，佤族、拉祜族、傈僳族等特困民族，直过民族、彝族支系俐侎人等特殊贫困群体进行重点扶持。深入实施兴边富民工程改善沿边群众生产生活条件三年行动计划，基本实现沿边地区群众“五通、八有、三达到”目标。加快推进新一轮民族团结进步示范点创建工程，新创建1个示范县、11个示范乡镇、100个示范村（社区）、1000户示范户。重视少数民族文化保护、传承和开发。注重少数民族人才培养，让每个少数民族人才都能成为率领本民族走向现代化的领路人。

　　（七）加快农村危旧房改造，在脱贫攻坚上实现重大突破。举全市之力打赢“27241”脱贫攻坚大会战，全面消除贫困，全面建成小康。坚持群众自主、自愿、自建原则，自上而下和自下而上相结合，以下为主、以村为主抓好规划，以县区为主统筹各类资金项目，杜绝大拆大建、打造亮点，确保到2018年全面完成19.3万户农村危房改造和抗震安居工程建设，让农村困难群众住上安居房。巩固和健全政府、市场、社会相互支撑，专项扶贫、行业扶贫、社会扶贫“三位一体”的大扶贫格局，全面落实扶贫目标、任务、资金、权责“四个到县”制度。坚持精准规划、精准施策，聚焦农村危房改造、改善基础设施、增加群众收入、提升群众素质、加强医疗救助、加强生态保护、社会保障兜底等工作重点，努力增强贫困群众自我发展能力。到2018年实现现行标准下农村贫困人口全部脱贫，8县区摘帽、27个贫困乡镇和241个贫困村出列。到2020年，稳定实现农村贫困人口不愁吃、不愁穿，义务教育、基本医疗和住房安全有保障，人均可支配收入增幅高于全市平均水平，基本公共服务主要领域指标接近全市平均水平。

　　（八）加快发展社会事业，在幸福临沧建设上实现重大突破。从解决人民群众最关心、最直接、最现实的利益问题入手，提高公共服务共建能力和共享水平，稳步增加城乡居民收入，使各族群众有更多获得感、幸福感。深化教育领域综合改革，基本普及学前教育，推进边境县实施14年免费教育；推进义务教育均衡发展，重视边远山区教育发展，科学规划校点布局；普及高中阶段教育，办好特殊教育，积极发展继续教育、民办教育；义务教育巩固率达95%以上，高中阶段毛入学率达90%以上，建成义务教育标准化学校1000所、省一级完中11所、特色优质中等职业学校9所。优化高等教育结构，支持滇西科技师范学院建成滇西名校、国门大学，支持职业高等教育加快发展。推进健康临沧建设，深化医药卫生体制改革，建立覆盖城乡的基本医疗卫生制度和现代医院管理制度，确保新农合参合率保持在98%以上。坚持中西医并重，加快发展中医药事业。切实加大重大传染病的防控，提高疾病预防控制能力。坚持计划生育基本国策，促进人口均衡发展。深化科技体制改革，持续推进临沧国家可持续发展实验区和低碳国土试验区建设，加快建设创新型临沧，力争研发投入占GDP比重达0.65%以上，公民具备基本科学素质比例达4.35%以上，科技进步对经济增长的贡献率达49%以上。大力发展文化体育事业，丰富各族群众文化生活。举办好全省第十五届运动会、第十一届残疾人运动会暨第五届特殊奥林匹克运动会、第十一届少数民族传统体育运动会、全省少数民族传统体育锦标赛和第九届农民运动会。积极促进就业创业和农村劳动力转移就业，努力实现城镇新增就业10万人以上，城镇登记失业率控制在4.5%以内。完善覆盖城乡的社会保障体系，实施“全民参保登记计划”，各项社会保险参保达214万人次左右，城乡居民基本养老保险参保率达99%以上。全面放开养老服务市场，养老床位达1万张以上。健全扶残助残服务体系，支持残疾人事业发展。加强社会治理，推进法治临沧建设，强化安全生产管理。加强食品药品安全监管工作，确保“舌尖上的安全”。高度关注边境动态，加快“智慧边境”建设，大力提升边境应急保障能力，管好、管住、管活边境，切实维护边境和谐安宁，创建平安临沧。

三、2016年工作安排

　　今年是全面建成小康社会决胜阶段的开局之年，做好今年的工作，对推动我市跨越发展具有十分重要的意义。我们要深入贯彻中央、省委、省政府和市委的决策部署，坚决按照“五位一体”和“四个全面”总体布局，牢固树立和贯彻落实“创新、协调、绿色、开放、共享”发展理念，打基础、补短板，调结构、建支柱，抓改革、促开放，拼命干、创佳绩，全力推进“四个一百”重点项目，全面完成27项重要指标任务，把“十三五”的龙头昂起来，确保开好头、起好步、开门红。

　　今年经济社会发展的主要目标建议为：

　　——生产总值增长11%以上；

　　——规模以上固定资产投资增长20%；

　　——规模以上工业增加值增长14%；

　　——一般公共预算收入增长5%；

　　——一般公共预算支出增长6%；

　　——社会消费品零售总额增长12%；

　　——外贸进出口总额增长15%；

　　——城镇常住居民人均可支配收入增长10%；

　　——农村常住居民人均可支配收入增长12%；

　　——招商引资实际到位资金增长15%；

　　——城镇登记失业率控制在4.5%以内；

　　——居民消费价格总水平累计涨幅控制在3%左右；

　　——单位生产总值能耗降幅1.4%。

　　为确保“十三五”良好开局，我们将着力抓好以下八个方面的工作：

　　（一）着力推进结构性改革，加快固定资产投资

　　稳妥推进供给侧改革。经济发展新常态，不等于不要发展，不等于不要GDP。临沧要实现跨越发展，必须在稳增长中推进结构性改革，把握稳增长与结构性改革的平衡，着力用结构性政策来稳定经济增长，在结构性改革中培育新的发展动能，提升改造传统比较优势，保证发展的质量和效益，保证发展的速度能快则快。要正确认识临沧发展优势和短板，明确好政策方向，错位竞争，不失时机推进供给侧结构性改革、产业结构调整，不断增加供给能力、提升供给能力的空间。要在适度扩大总需求的同时，从生产领域加强优质供给，减少无效供给，扩大有效供给，提高供给结构适应性和灵活性，提高全要素生产率，使供给体系更好适应需求结构变化。要按照国家、省供给侧结构性改革方案的部署要求，多措并举化解过剩产能，化解房地产库存，主动稳妥“去杠杆”，打好降本增效组合拳，全力补齐发展短板。要紧紧盯住今年稳增长的各项目标任务，落实好各项稳增长的政策措施，保持换挡不降速的定力，确保在稳增长中推进结构性改革取得新成效。

　　狠抓固定资产投资。临沧的经济发展总量、规模和水平，决定了投资仍然是临沧今后一段时期经济社会持续快速发展的最主要推动力量。必须紧紧抓住固定资产投资，扎实推进“四个一百”重大建设项目，强化土地、财税、融资等要素保障，确保新开工项目加快建设，在建项目尽快竣工，竣工项目迅速投入使用产生效益。要加大向上争取力度，争取更多的项目进入国家和省相关规划，争取更多中央和省投资向临沧集聚，完成向上争取资金173亿元。要加快项目储备和前期，完善项目储备库，加大项目前期工作经费投入，加快引进一批对全市经济社会发展牵动力强的大企业、大项目，加快谋划开工一批重大项目。要创新投融资体制，发挥投融资平台作用，积极推行政府与社会资本合作（PPP）投资模式，千方百计增加项目投资。要深化银政企合作，争取扩大信贷规模，培育各类财源，严格控制一般性财政支出，加大对重大项目建设的保障力度，完成一般公共预算收入40亿元以上，完成一般公共预算支出214亿元以上，市内金融机构新增融资50亿元以上。要用好地方债发行政策，以及特许经营、合理定价、政府补贴等政策，加强政府债务管理及风险预警，守住不发生系统性区域性金融风险底线。

　　（二）着力加快“五网”基础设施建设，尽快补齐发展“短板”

　　加快推进综合交通重大项目建设。全力以赴抓好大临铁路征地拆迁和建设，完成投资15亿元。加快玉临高速公路征地拆迁，实现境内段全面开工，完成投资20亿元。加快机场高速公路建设，完成投资10亿元。开工建设临沧至清水河高速公路及临沧城连接线、国道219线临沧境内段改扩建项目。加快推进3条（段）沿边二级公路、德党至勐堆二级公路、云凤一级公路、澜沧江沿江公路建设，沿边二级公路小黑江桥至勐省段基本建成。完成临沧至清水河铁路和大理至临沧、云县至保山高速公路前期工作，启动大理（巍山）至凤庆（鲁史）至永德（永康）公路改造项目前期工作。全面完成1680公里建制村通畅工程和11座溜索改桥项目，新开工1637公里建制村通畅工程。确保沧源佤山机场10月建成通航，加快临沧机场改扩建工程，扎实推进孟定机场和凤庆、永德通用机场前期工作。力争完成综合交通投资120亿元。

　　加快解决工程性缺水问题。2月底开工建设耿马灌区。开工建设耿马团结、云县岔河2件中型水库，3件小（一）型水库。加快推进在建的永德德党河、大雪山、镇康中山河、临翔鸭子塘、双江韭菜坝、沧源东丁、云县晓街河、耿马十八道河、凤庆郭大寨等9件中型及20件重点小（一）型水库建设。确保竣工1件中型、4件小（一）型水源工程。力争完成蓄水3.3亿立方米。

　　加快推进能源保障建设。推进220千伏博南Ⅱ回线工程建设，不断完善临沧电网220千伏网架结构，加快电力枢纽工程和电网设施建设，确保骨干电网、城市配网建设取得新进展。规范发展水电产业，力争投产水电站装机22.3万千瓦。加快天然气保障工程建设，力争临翔区、凤庆县、云县城市天然气项目建成通气，启动其他县城天然气建设项目。

加快建设信息保障工程。落实好“中国制造2025”云南行动计划以及“两化”深度融合专项行动计划，实施“智慧临沧”、“宽带临沧”、“互联网+”行动计划。启动建设滇西信息产业园、特色农产品和孟定滇缅特色产品电子商务物流中心。加快农村电子商务平台建设。

　　（三）着力推进务实合作，努力促进跨境“人财物”三通

　　加快推进对缅互联互通工程。全力推动滚弄大桥援建项目获批并开工建设，加快推动清水河至腊戍出境铁路建设纳入国家战略规划。继续推进边境地区供水、电力、通信等基础设施互联互通。以适当方式在缅甸内比都、仰光、曼德勒、东枝、腊戍设立临沧商务代表处，成立中国国际贸易促进会临沧分会。提高通关便利化水平，加快推进清水河口岸迁址新建，加快推进“一口岸、多通道”模式创新，做好南伞口岸、永和口岸升格为一类口岸申报工作，积极推动缅方清水河口岸持护照通行、跨境游异地办证获批，启动运行3条边境旅游线路。抓好清水河粮食进口指定口岸管理，加快入境种苗、水果、水产品等农产品指定口岸申报工作，抓好国家坚果类检测重点实验室建设，争取将临沧列为解决非法入境偶蹄动物问题试点。以“企业、高端、合作”为特色，突出缅甸元素，办好第六届临沧边境经济贸易交易会。

加快推进边合区建设。认真贯彻落实《国务院支持沿边重点地区开放若干政策措施的意见》，伺机启动边合区体制机制改革。启动综合保税区规划申报工作，积极推动南伞—老街跨境经济合作区建设，争取国务院批准设立镇康—果敢跨境工业园区。加快推进清水河口岸联检大楼、查验货场、主道路建设。进一步推进大额人民币现钞出入境携带证管理业务和跨境支付服务业务，推动建立外汇特许兑换业务，畅通中缅边境人民币结算、清算渠道。积极承接加工贸易向边合区梯度转移，努力实现规模以上固定资产投资242.4亿元，招商引资实际到位资金278.2亿元，外贸进出口总额40.4亿元，规模以上工业增加值20.8亿元。

　　深化全方位交流合作。加强与斯里兰卡加姆波勒市、俄罗斯杜布纳市等友好城市的交流合作。深入推进与长江流域、成渝经济区和滇中地区、滇西周边地区的交流合作，加强与泛珠三角、长三角、环渤海、京津冀和港澳台等发达地区合作。不断优化招商环境，创新招商方式，提高招商质量，实现招商引资实际到位资金955亿元。

　　（四）着力提升生物产业，促进“两型三化”发展

　　加快发展生物产业。扶持农民发展优质农产品生产基地，支持农业龙头企业开展示范基地、园艺作物标准园、畜禽养殖标准化示范场、水产健康养殖示范场等标准化生产基地建设。在农产品精深加工上下功夫，启动建设滇西生物产业园，推进生物资源精深加工集聚发展。在核桃、蔗糖等行业加大招商引资力度，促进产业升级和持续发展。完成中低产田地改造23.16万亩。培育10户年销售收入达5亿元以上涉农企业“小巨人”，新建100个农民专业合作社，培育发展100个家庭农场。确保粮食总产量105万吨，茶叶产量11.5万吨，收购烤烟85.4万担。

　　积极培育工业经济增长点。加快建设临沧工业园区、云县新材料光伏产业园区两个百亿元园区，推进全国滇红茶知名品牌创建示范区和其他县域工业园区建设，园区规模以上工业增加值占全市比重65%以上。加快科技创新园、科技孵化园建设，促进“大众创业、万众创新”。继续开展服务企业年活动，注重帮助企业降低成本，提高效益。重点抓好100个规模以上项目，确保全市工业固定资产投资增长10%以上，规模以上工业增加值达104.7亿元，全社会用电量达24.8亿千瓦时，增长10%以上。坚持政府、企业、蔗农共渡难关，巩固提升蔗糖产业，加快推进88万亩糖料蔗核心基地建设，在高产糖料基地建设、蔗和糖产品开发加工上取得突破。做好临沧打叶复烤厂建设项目各项前期工作，争取尽快开工建设。大力支持民营经济加快发展，继续推进“两个10万元”微型企业培育工程，力争民营经济增加值增长11%以上。

　　加快发展现代服务业。提高第四届亚洲微电影艺术节规模、水平、档次。办好首届中国临沧凤庆国际红茶节和云南省航空旅游市场推介会。以沧源国际旅游度假区和滇西南生态养生旅游圈建设为重点，加快打造旅游景点，开展好沧源“摸你黑”狂欢节、“澜沧江啤酒”狂欢节等节庆活动，确保旅游总收入增长25%。以发展对缅物流业为重点，加快建设中缅边境商贸物流、出口加工基地。继续支持限额以上批零住餐企业及个体户发展，确保批发、零售业商品销售额增长15%，住宿、餐饮业营业额增长17%。加快融入全省“云上云”行动计划，积极发展连锁经营、物流配送、电子商务等新型流通方式，工业企业电子商务销售额增长30%以上。

　　（五）着力提高城镇规划建设管理水平，加快推进新型城镇化

　　抓好城乡规划建设。结合铁路、高速公路过境布局，加快规划建设临沧城东片区，开发利用好低丘缓坡地区，促进临沧中心城市扩容提质。高起点，高标准，启动规划建设孟定新兴口岸城市。加快县城建设，统筹沿边地区、沿澜沧江地区、沿南汀河地区城镇建设，引导城镇组团发展。加快推进续建的8个省级城市综合体建设，抓好城市地下综合管廊、地下空间开发利用规划建设。加快建设城镇道路、供水、供电、绿化、停车场、公厕、“两污”处理等配套设施，注重旧城区改造提升，不断完善城市功能。启动和完成乡镇总体规划修编，以及控规、专项规划、近期建设规划编制工作。全市不在县城规划区范围内的66个乡镇，实行县区领导挂钩责任制，努力打造一批集生态旅游、商贸加工为一体的特色小镇，切实解决好乡镇“不漂亮、不洁净、无特色”的问题。继续推进耿马县中小城市综合改革试点和低丘缓坡土地综合利用试点。抓好28个被认定的中国传统村落保护利用工作，争取一批村庄列入国家保护名录，建成一批美丽宜居乡村。

　　提高城市管理水平。以标准化管理为基础，以网格化为支撑，以依法管理贯穿始终，建立社会组织参与的“精细化管理”城市管理网络，加强市容市貌管理，规范临街牌匾，集中整治户外广告、非法张贴、占道经营、私搭乱建、车辆乱停乱放等行为。推进住宅小区规范管理，提高物业管理水平。坚持“纵向到底、横向到边、不留死角”，加大环境卫生管理力度，让市民生活更方便、更舒适、更健康。

　　科学推进产城融合。加快发展特色县域经济和城镇经济，推动城镇化由产业造城向产业兴城转变，由宜业向宜居宜业兼顾转变。抓好住房公积金管理工作，切实为职工解决住房问题。引导房地产业健康发展，积极支持合理的住房消费需求，商品房销售面积达65万平方米以上。

　　积极引导农民进城。加快保障性住房续建工程建设，提高保障性住房入住率。落实棚户区改造三年行动计划，实施棚户区改造2.26万户，基本建成棚户区改造、公共租赁住房1万套，提高货币化安置比例。深化户籍制度改革，维护进城农民土地承包、宅基地使用、集体收益分配权益，保障进城农民享受城镇基本公共服务，有序推进农业转移人口市民化，城镇化率达38%以上。

　　（六）着力加强生态保护和建设，提高生态文明水平

　　加快推进生态建设。科学划定生态保护红线，制定重点生态功能区产业准入负面清单，积极推进国家主体功能区建设试点示范。加强沿江、沿路、沿边“三线”重点区域生态系统的保护和修复，改善区域生态景观，保护生物多样性。完成新造林74万亩、退耕还林21.37万亩、荒山造林2500万株，陡坡地生态治理2万亩，森林覆盖率提高到68.5%。更加注重水生态系统保护，开展临翔区水生态文明建设试点。启动实施生态清洁型小流域综合治理工程，新增水土流失治理面积290平方公里。加强农村饮用水水源地保护，完成日供水1000吨或服务人口1万人以上的饮用水水源地保护区划定工作。完成415个“市级生态文明村”创建命名和52个“省级生态文明乡镇”创建申报工作。

　　积极推行低碳循环发展。支持绿色清洁生产，推动发展绿色低碳循环产业体系，鼓励企业加快能源技术创新，推进燃煤工业锅炉节能环保综合改造，重点在制茶行业推广太阳能利用技术和装备。淘汰和化解过剩产能，确保年度能源消耗总量、单位GDP能耗和碳排放量、主要污染物排放总量目标全面落实。

　　全面加强环境整治。深入开展“洁净临沧”行动，积极创建国家卫生城市。推进重点流域、重点区域和各类园区规划环评，全面实施大气污染防治计划，继续加强主要过境河流生态整治，注重农村环境集中连片综合整治，加大重金属污染风险企业日常监管力度，有效防范环境污染事件。

　　（七）着力推进农村危旧房改造，加快实施精准脱贫

　　坚持精准施策、精准扶贫。统筹推进农村危房改造、抗震安居工程、地震恢复重建、易地扶贫搬迁工程，抓好水利水电移民搬迁安置和后续产业发展，完成农村危旧房改造和抗震安居工程6.46万户。致力于贫困乡村道路、电网、农田水利、安全饮水、通讯、广播电视、医疗卫生、基础教育设施和农村集贸市场建设，改善贫困群众生产生活条件，增强贫困群众自我发展能力。实现云县脱贫摘帽，14个贫困乡镇、130个贫困村出列，减少贫困人口8.59万人。

　　扶持民族地区加快发展。实施新一轮民族团结进步示范点创建工程、兴边富民工程改善沿边群众生产生活条件三年行动计划、新一轮扶持彝族支系俐侎人发展三年专项规划、扶持人口较少民族发展和特困区域扶贫开发等工程，加快消除人口较少民族聚居区危旧房，提高民族地区群众收入水平、人均受教育年限和公共服务程度。

　　（八）着力保障和改善民生，促进社会和谐稳定

　　提升就业水平。实施“贷免扶补”、创业担保贷款、劳动密集型小企业贷款、微型企业培育工程，以创业带动就业。突出抓好高校毕业生就业工作，统筹做好城镇困难人员、农村转移劳动力、退役军人等各类群体就业工作。实现城镇新增就业2.5万人以上，农村转移就业15万人次以上。

　　增强社会保障。推进机关事业单位养老保险制度改革，全面实施“全民参保登记计划”，完成五项社会保险参保200万人次以上的参保扩面目标，确保新农合参合率保持在99%以上。建立健全养老服务业发展的配套政策，稳步提升千名老人床位比例。不断完善新型城乡社会救助体系，加快提高弱势群体生活水平。

　　增加城乡居民收入。大力发展实体经济，不断增加居民经营性收入。保护农民工合法权益，推行企业工资集体协商制度，完善最低工资增长机制，稳步增加居民工资性收入。积极推进不动产统一登记改革，多渠道增加居民财产性收入。持续增加居民转移性收入。积极稳妥改革分配制度，缩小城乡、行业收入差距。

　　统筹推进社会事业发展。全面完成沧源“3·01”地震灾后恢复重建。继续深化教育综合改革，重视农村基础教育，优化农村边远山区、少数民族地区校点布局，推进边境县实施14年免费教育。加快发展学前教育和高中阶段教育，学前教育三年毛入园率达49%，高中阶段毛入学率达72%。推进义务教育均衡发展，建成义务教育标准化学校200所，义务教育巩固率达91%。确保临翔区、云县、凤庆县、双江县义务教育均衡发展通过国家验收。努力提高滇西科技师范学院、临沧开放学院办学规模和质量。继续推进以县级公立医院为重点的医药卫生体制改革，加快发展中医药事业，加强重大传染病防控，提高基层公共卫生服务能力。落实一对夫妇可生育两个孩子政策。持续增加科技投入，加大科技成果转移转化力度。加快建设一批文化体育设施，健全城乡公共文化服务体系，确保市博物馆、文化馆、图书馆建成投入使用，启动建设市档案馆、市老年文化体育活动中心，统筹推进老年体育事业发展，广泛开展群众体育健身活动，举办好全省第九届农民运动会。支持驻临军警部队现代化建设、国防动员和民兵预备役工作，推进军民融合深度发展，创建全国双拥模范城。支持工会、共青团、妇联、科协、工商联、社科联、文联、关工委、妇儿工委做好工作。支持红十字会、老龄、慈善、残疾人等事业健康发展。抓好统计调查工作，开展好第三次全国农业普查。加强工商、质监、供销、粮食、地方志、侨务、外事、人防等工作。推进气象、地质防灾减灾体系建设，提升防震、水文服务能力。

　　加强和创新社会治理。深入推进依法治市，创新社会治理方式。认真开展“法律六进”、“七五”普法工作。扩大援助范围，提高援助质量，保证人民群众获得及时有效的法律帮助。有效预防和化解社会矛盾，推进信访法治化。高度重视公共安全问题的预防、预警、反应和处置，建立健全社会治安、食品安全、网络安全、安全生产、防灾减灾等领域公共安全体系和突发公共事件应急体系。加强社会治安综合治理，扎实开展禁毒防艾人民战争。依法打击邪教组织和各类违法犯罪活动。坚持不懈抓好边境维稳处突能力建设和边境文明执法活动，确保边境和谐稳定。

　　认真实施10件惠民实事。

　　各位代表！“十三五”和新一年的艰巨任务，是全市各族人民的重托和期望，我们深感使命光荣、责任重大。我们要以对党和人民的忠诚，对理想和信念的执着，对临沧的热爱和奉献，用“三严三实”和谋事创业的担当，切实转变作风，提升能力，认真履职，以舍我其谁的信心和勇气，拼命干，干成事，向全市人民交上一份满意的答卷。

　　——加快转变政府职能。推进行政审批方式创新，最大限度减少自由裁量权。凡是市场机制能有效调节的经济活动，一律取消审批，由基层管理更方便有效的经济社会事项，一律下放基层管理。推进审批事项授权办理制、重点项目并联审批制、企业重大项目代办制、三级联动审批工作制，促进行政审批提速增效。着力建设高效的行政审批网上服务大厅，方便群众和企业办事。改革完善财政管理制度，积极稳妥推进政府向社会购买服务工作，增强政府提供公共服务和公共产品的能力。

　　——加快建设法治政府。认真贯彻《法治政府建设实施纲要（2015—2020年）》，制定好政府各类规章制度，做到法无授权不可为、法定职责必须为。深化行政执法体制改革，建立权责统一、权威高效的依法行政体制，推进行政机关职能、权限法定化。严格执行重大执法决定法制审核制度，建立健全依法行政责任追究制度。健全依法决策机制，全面推行政府法律顾问制度。重视政府新型智库建设，提高科学决策水平。全力推进政府权力清单、责任清单和投资企业负面清单制度实施，规范行政权力运行。加快社会诚信体系建设，推进政务公开，逐步扩大行政决策公开的领域和范围。

　　——切实转变工作作风。发扬心中有民、怀揣理想，顶天立地、敢为人先，坚韧拼搏、满腔热情，想干事、能干事、干成事的“临沧作风”，创造“临沧速度”“临沧效率”。强化党风廉政主体责任，进一步巩固“三严三实”专题教育成果，认真开展“两学一做”学习教育，坚决反“四风”。完成公务用车制度改革。全面提升各级政府和干部队伍深化改革、推动发展、化解矛盾、维护稳定的能力，全面提高驾驭市场经济、领导经济工作和推动现代化建设的水平。增强干部创新意识、担当意识、风险意识，集中精力抓落实，齐心协力促跨越。努力做到干部清正、政府清廉、政治清明，营造风清气正、干事创业的从政环境。

　　——加强监督问责。继续加强工作绩效考核，完善奖惩机制，坚决整治乱作为、懒作为、不作为，不用心、不尽责，做“太平官”等行为；严格问责抓工作不力，落实中央、省委、省政府和市委、市政府工作部署不力的部门和干部。自觉接受市人大及其常委会的法律监督、工作监督，市政协的民主监督，新闻舆论和社会公众的监督，认真办理人大代表批评、意见、建议和政协委员提案。高度重视民主党派、工商联和无党派人士的意见、建议。进一步加强行政监察和审计监督。

　　各位代表！时代赋予重托，奋斗铸就辉煌。让我们紧密团结在以习近平同志为核心的党中央周围，在市委的坚强领导下，踏石留印、抓铁有痕，不屈不挠、开拓前进，在主动服务和融入国家战略中实现临沧跨越发展，创造临沧更加美好的明天！
